# Trust in science and scientists among university students, staff, and faculty of a large, diverse university in Los Angeles during the COVID-19 pandemic, the Trojan Pandemic Response Initiative

**DOI:** 10.1186/s12889-023-15533-x

**Published:** 2023-03-30

**Authors:** Michele Nicolo, Eric Kawaguchi, Angie Ghanem-Uzqueda, Daniel Soto, Sohini Deva, Kush Shanker, Ryan Lee, Frank Gilliland, Jeffrey D. Klausner, Lourdes Baezconde-Garbanati, Andrea Kovacs, Sarah Van Orman, Howard Hu, Jennifer B. Unger

**Affiliations:** 1 Department of Population and Public Health Sciences, Keck School of Medicine, University of Southern California, Los Angeles, CA USA; 2 Department of Nutrition and Food Science, California State University, Los Angeles, CA USA; 3 Family Medicine, Keck Medicine of USC, Los Angeles, CA USA; 4 Keck School Medicine of USC, University of Southern California, Los Angeles, USA; 5 University of Southern California, SSB 302, 2001 N. Soto Street, Los Angeles, CA 90033 USA

**Keywords:** COVID-19, SARS-COV-2, College, University, Trust in science inventory

## Abstract

**Background:**

Mistrust in science and scientists may adversely influence the rate of COVID-19 vaccination and undermine public health initiatives to reduce virus transmission.

**Methods:**

Students, staff and faculty responded to an email invitation to complete an electronic survey. Surveys included 21-items from the Trust in Science and Scientists Inventory questionnaire. Responses were coded so higher scores indicated a higher trust in science and scientists, A linear regression model including sex, age group, division, race and ethnicity, political affiliation, and history of COVID-19, was used to determine variables significantly associated with trust in science and scientists scores at the p < 0.05 level.

**Results:**

Participants were mostly female (62.1%), Asian (34.7%) and White (39.5%) and students (70.6%). More than half identified their political affiliation as Democrat (65%). In the final regression model, all races and ethnicities had significantly lower mean trust in science and scientists scores than White participants [Black ($B$= -0.42, 95% CI: -0.55, -0.43, p < 0.001); Asian ($B$= -0.20, 95% CI: -0.24, -0.17, p < 0.001); Latinx ($B$= -0.22, 95% CI: -0.27, -0.18, p < 0.001); Other ($B$= -0.19, 95% CI: -0.26, -0.11, p < 0.001)]. Compared to those identifying as Democrat, all other political affiliations had significantly lower mean scores. [Republican ($B$ =-0.49, 95% CI: -0.55, -0.43, p < 0.0001); Independent ($B$ =-0.29, 95% CI: -0.33, -0.25, p < 0.0001); something else ($B$ =-0.19, 95% CI: -0.25, -0.12, p < 0.0001)]. Having had COVID-19 ($B$= -0.10, 95% CI: -0.15, -0.06, p < 0.001) had significantly lower scores compared to those who did not have COVID-19.

**Conclusion:**

Despite the setting of a major research University, trust in science is highly variable. This study identifies characteristics that could be used to target and curate educational campaigns and university policies to address the COVID19 and future pandemics.

## Background

Misinformation about COVID-19 and mitigation practices including vaccination has proliferated throughout the pandemic, potentially increasing distrust of science and scientists [1, 2]. Conflicting guidelines and practices for mitigating the spread of the virus (e.g., changing mask mandates) at the state and federal levels in the U.S. produced confusion among the population often resulting in poor health outcomes [3–7]. Wariness of the science surrounding COVID-19 has sparked controversy over safety of the COVID-19 vaccine and increasing hesitancy among certain subgroups in the population [8–11]. Vaccine hesitancy and denial are likely responsible for a large portion of the estimated 319,000 excess COVID-19 deaths (as of April 30, 2022) that vaccinations could have been prevented in the U.S. [12].

Mistrust in science and scientists is embedded in history especially among certain racial and ethnic groups [9] as a product of structural racism and health disparities [13–15]. While public health messaging around the COVID-19 vaccine has targeted underserved communities in the U. S. [16, 17], the percentage receiving at least one dose of the vaccine among those who identified as Black (57%) is less than those identifying as White (63%) and those identifying as Hispanic (65%).[18] Religious beliefs and political affiliation have been associated with suspicion surrounding COVID-19.[19–22] Furthermore, political affiliation influences individuals’ selection of health information sources, leaving room for misinterpretation of scientific recommendations on mitigation practices [23]. Few studies have investigated trust in science and scientists among students, staff and faculty within a university [24, 25]. Completing more college level science based courses (8 or more) is associated with greater trust in science compared to taking fewer courses (2 or less). [26] Better understanding of science is considered a factor in greater trust in science and scientists [24], however, it is not clear whether characteristics of individuals attending and working in an academic environment reflect similar characteristics associated with trust in science among populations in the U.S. Furthermore, data is lacking whether trust differs among students and faculty based on academic interests for example political science and religious studies. Therefore, the aim of this study is to investigate characteristics associated with trust in science and scientists among students, staff, and faculty attending or working at a large, diverse, urban university.

## Methods

### Participants

Participants were students, staff, and faculty at the University of Southern California (USC) in Los Angeles, California, a major R-1 research-intensive University. Participants were eligible if they were currently employed or enrolled at USC, were at least 18 years of age, and provided informed consent completed online.

### Procedure

This study was approved by the USC Institutional Review Board. Methods and procedures have been published elsewhere [8]. Briefly, all graduate and undergraduate students enrolled at the university in addition to staff and faculty were invited by email to participate in a brief COVID-19 survey. All student, faculty and staff with an institutional email received survey invitations. Duplications were removed based on participant university identification number and date of birth. Participants with completed online consent forms were enrolled. Survey responses from enrolled students, staff and faculty with a university email and completed online consent form were included. Responses were collected from 4/29/2021 to 4/22/2022.

### Outcome variable

Trust in science and scientists scores were calculated using responses to the Trust in Science and Scientists Inventory, a validated 21-item questionnaire [2, 26–29]. Responses to each item ranged from 1” strongly disagree” to 5 “strongly agree”. Items were coded so that a higher score indicated a higher trust in science and scientists, and the 21 items were averaged together to form a single Trust in Science and Scientists score (Cronbach’s alpha = 0.86).[26] Of the number of survey respondents (N = 9684), only participants who completed the Trust in Science and Scientists Inventory were included in the analyses (N = 4237) since this was the outcome variable of interest.

### Covariates

Demographic variables included self-reported sex (male, female), race/ethnicity (Black, Asian, Latinx, Other race and ethnicities and White), division (student, staff, and faculty) and age. “Other” race and ethnicity category included participants identifying as multi-racial or as a race/ethnicity other than Black, Asian, Latinx or White. Greater than 80% of students were between ages 18 and 24 years, and ages among staff and faculty ranged between 18 and 85 years. To further investigate whether age is associated with trust in science and scientists, age was categorized into quartiles. Political affiliation (Republican, Democrat, Independent or something else) and self-reported COVID-19 infection status was categorized as “yes” or “no”.

### Data analysis

All analyses were performed using STATA version 17.0 (StataCorp, USA). P-values for all analyses were two-sided with a significance level of $\alpha =0.05$. A multiple linear regression model including all covariates (race and ethnicity, age category, sex, division, political affiliation, and previous COVID-10 status) were entered in a stepwise manner to determine which variables were associated with trust in science and scientists scores. Multicollinearity was checked using the Variance Inflation Factor for each independent variable inputted into the model. Beta coefficients and 95% confidence intervals associated with mean trust in science and scientists score are reported.

## Results

Demographic characteristics and mean trust in science and scientist scores are displayed in Table 1. The sample was primarily female (62.1%), Asian (34.7%) and White (39.5%) and were students (70.6%). More than half identified their political affiliation as Democrat (65%). Participants identifying as Black had the lowest mean trust compared to other races and ethnicities (p < 0.0001). Males (v. females; p = 0.002) and those reporting having had COVID-19 (v. not reporting COVID; p < 0.0001) also had lower mean trust in science and scientist scores. Participants reporting political affiliation as Republican had lower scores compared to other affiliations (p < 0.0001).

**Table 1:** Demographic characteristics and mean attitude towards science/scientists

Variable	N (%)	Attitudes towards science M(SD)	p-value
Sex			
Male	3671 (37.9)	3.87 (0.56)	**0.002**
Female	6013(62.1)	3.92 (0.52)	
Race and ethnicity			
Black	446 (4.6)	3.64 (0.61)	**< 0.001**
Asian	3346 (34.7)	3.81 (0.51)	
Latinx	1549 (16)	3.83 (0.55)	
Other	487 (5.0)	3.83 (0.56)	
White	3825 (39.5)	4.04 (0.50)	
Age category (years)			
18–20	2507 (25.9)	3.92 (0.49)	**< 0.001**
21–24	2622 (27.1)	3.85 (0.55)	
25–32	2165 (22.4)	3.92 (0.55)	
33–85	2389 (24.7	3.91 (0.55)	
Division			
Staff/faculty	2845 (29.4)	3.91 (0.54)	0.34
Student	6838 (70.6)	3.89 (0.52)	
Had COVID			
Yes	572 (13.5)	3.79 (0.59)	**< 0.001**
No	3653 (86.5)	3.92 (0.52)	
Political affiliation			
Republican	449 (6.6)	3.52 (0.56)	**< 0.001**
Democrat	4438 (65)	4.00 (0.49)	
Independent	1495 (21.9)	3.70 (0.54)	
Something else	449 (4.6)	3.81 (0.58)	

^A^ Independent Student t-test

^B^ Analysis of Variance

M: mean; SD: Standard Deviation; %: percentage; N: number

Multivariate linear regression results are shown in Table 2. All races and ethnicities were significantly lower than White participants [Black ($B$= -0.42, 95% CI: -0.55, -0.43, p < 0.001); Asian ($B$= -0.20, 95% CI: -0.24, -0.17, p < 0.001); Latinx ($B$= -0.22, 95% CI: -0.27, -0.18, p < 0.001); Other ($B$= -0.19, 95% CI: -0.26, -0.11, p < 0.001)]. In a post hoc analysis, trust in science and scientists scores among Black participants were significantly lower than Asian (p = 0.002); Latinx (p = 0.009) and Other races and ethnicities (p = 0.012).

**Table 2:** Correlates of attitudes towards science/scientists using multiple linear regression analyses

Variable	Coefficient (SE)	95% CI	p-value
Sex			
Male ^A^			
Female	0.01 (0.01)	-0.22, 0.08	0.54
Division			
Staff ^A^			
Student	0.03 (0.02)	-0.01, 0.08	0.17
Race/ethnicity			
Black	-0.42 (0.04)	-0.55, -0.43	**< 0.0001**
Asian	-0.20 (0.01)	-0.24, -0.17	**< 0.0001**
Latinx	-0.22 (0.02)	-0.27, -0.18	**< 0.0001**
Other	-0.19 (0.03)	-0.26, -0.11	**< 0.0001**
White ^A^			
Age category (years)			
18–20	-0.01 (0.03)	-0.07, 0.05	0.71
21–24	-0.07 (0.03)	-0.13, -0.01	**0.01**
25–32	-0.01 (0.02)	-0.50, 0.04	0.84
33–85 ^A^			
Political affiliation			
Democrat ^A^			
Republican	-0.49 (0.03)	-0.55, -0.43	**< 0.0001**
Independent	-0.29 (0.01)	-0.33, -0.25	**< 0.0001**
Something else	-0.19 (0.03)	-0.25, -0.12	**< 0.0001**
Had COVID-19			
Yes	-0.10 (0.02)	-0.15, -0.06	< 0.**0001**
No ^A^			

Multiple Linear Regression Analysis

^A^ Reference category

SE: Standard Error; %: percentage

CI: Confidence Interval

All political affiliations [Republican ($B$ =-0.49, 95% CI: -0.55, -0.43, p < 0.0001); Independent ($B$ =-0.29, 95% CI: -0.33, -0.25, p < 0.0001); something else ($B$ =-0.19, 95% CI: -0.25, -0.12, p < 0.0001)] compared to Democrat, and having COVID-19 ($B$= -0.10, 95% CI: -0.15, -0.06, p < 0.001) were associated with lower mean trust in science and scientist scores. Only ages 21–24 years had lower scores compared to those in the oldest quartile for age (($B$ =-0.07, 95% CI: -0.13, -0.01, p = 0.01). Sex and division were not significant correlates of trust.

## Discussion

This study investigated characteristics associated with trust in science and scientists among a sample of students, staff, and faculty at a large diverse university. Results from the analyses identified sex, race and ethnicity, age, political affiliation, and COVID-19 infection status are significantly associated with trust in science and scientists. Previous studies suggested university students are more inclined to value science compared to the general population and therefore have greater trust in science and scientists [27, 30]. Conversely, results from this study suggest characteristics associated with trust in science and scientists may be more aligned with results published in the literature which are based on general population characteristics, and affiliation with an academic institution may be less influential.

In the present study race and ethnicity was significantly associated with trust in science and scientists. These results are consistent with studies highlighting the effect of historical and structural racism against African American and Native American populations in the United States [15, 31–33]. Higher education institutions must be considerate of mistrust in science due to structural racism among various ethnic groups on campus.

Only scores among participants ages 21–24 were significant compared to older and younger adults. These results differ from those published by Dohle et al. who conducted a cross-sectional study (N = 962 adults) investigating the association between trust in science and acceptance of COVID-19 preventive measures during spring of 2020. The authors did not find a significant association between age and trust in science; however, the authors included age as a continuous variable rather than categorizing age to determine difference among age groups. We included division in the adjusted model since greater than 80% of student ages were between 18 and 24 years, yet, found lower trust scores among this age group. Because university campuses are highly interactive communities, understanding characteristics associated with complying with COVID-19 mitigation measures to needs further investigation.

Several studies have found political affiliation is a strong predictor of adopting COVID-19 prevention measures such as social distancing and mask wearing [2, 8, 22, 23, 34, 35]. COVID-19 misinformation delivered through different social and news media platforms aligning with certain political ideations have been found to influence acceptance of government public health messaging regarding mitigation measures including vaccination [9, 13, 22]. Despite being a public health crisis, COVID-19 has been politicized negatively impacting new infection and vaccination rates in the United States. This study found trust in science and scientists scores significantly differed among political affiliations with Democrats having the highest scores. Over 60% of the sample in this study identified as Democrats which is greater than reported Democratic political party affiliation, 46.5%, among registered voters in California in 2021 [36]. Further research on health outcomes related to the association of public health messaging and political ideation is needed to improve public health initiatives among individuals with lower trust in scientific based interventions.

Having COVID-19 was observed as a significant factor influencing lower trust scores. Perceived risk level to the virus may have influenced whether individuals were adherent to COVID-19 prevention measures. It is possible those who perceived themselves at higher risk may have adopted prevention measures compared to those who considered themselves to be less at risk. We did not include self-reported COVID-19 prevention behaviors in these analyses; therefore, it is unclear whether trust scores may have been moderated by perceived risk.

The current study adds to the literature in several ways. Survey responses were obtained from a large, diverse sample of students, staff, and faculty from a large research-intensive university in Southern California. Furthermore, our findings suggest trust in science and scientists are influenced by several factors including race and ethnicity, having COVID, and political affiliation. Although results correspond with other published literature, to our knowledge this is the first study to investigate trust in science and scientists among university students, staff, and faculty.

## Limitations

This analysis was based on a non-random sample of university students, staff, and faculty who responded to an online survey, and results may not generalize to the larger population. Future studies are needed to investigate correlates of trust in science and scientists among students, staff, and faculty who chose not to participate. Another limitation is self-reported data including COVID-19 infection status. Although we did ask about international student status, greater than 80% of the respondents were domestic students; therefore, we are unable to determine whether scores differed among domestic and international students.

## Conclusions

This study identifies factors associated with trust in science and scientists among university students, staff, and faculty including race and ethnicity, age, division within the university (student versus staff and faculty) and political affiliation. Furthermore, this study provides insight into characteristics influencing trust in science and scientists and identify populations that may benefit from targeted education and outreach campaigns. Additionally, these findings may help generate university policies and programs to address future pandemics. More research on the influence of public health messaging and political ideation on health outcomes especially among those with decreased trust in science is needed.
